# Modeling Soil Organic Carbon Change across Australian Wheat Growing Areas, 1960–2010

**DOI:** 10.1371/journal.pone.0063324

**Published:** 2013-05-16

**Authors:** Guocheng Wang, Yao Huang, Enli Wang, Yongqiang Yu, Wen Zhang

**Affiliations:** 1 State Key Laboratory of Atmospheric Boundary Layer Physics and Atmospheric Chemistry, Institute of Atmospheric Physics, Chinese Academy of Sciences, Beijing, China; 2 State Key Laboratory of Vegetation and Environmental Change, Institute of Botany, Chinese Academy of Sciences, Beijing, China; 3 University of the Chinese Academy of Sciences, Beijing, China; 4 Land and Water, Commonwealth Scientific and Industrial Research Organisation, Canberra, Australia; DOE Pacific Northwest National Laboratory, United States of America

## Abstract

Soil organic carbon (SOC) dynamics in Australian wheat-growing areas were simulated from 1960 to 2010 using Agro-C, a calibrated and validated biogeophysical model. Previously published data from field measurements were used to parameterize the Agro-C model. Model simulations show a decreasing trend in SOC over the last 50 years, mainly attributable to relatively low organic carbon (C) inputs. The rate of decrease in SOC tended to slow in the last two decades due primarily to an increase in wheat yields, which resulted in an increase in C input. Overall, we estimate that Australian wheat-growing areas, covering an area of 15.09 million hectares (Mha), lost 156 (86–222, 95% confidence interval) Tg C in the topsoil (to 30 cm depth) from 1960 to 2010. Approximately 80% of the SOC loss occurred in the period between the 1960s and the 1980s. Spatially, the SOC loss in areas with relatively high temperature and low precipitation, such as Queensland, the northern part of New South Wales and Western Australia, was more significant than that in other areas. We suggest that the loss of SOC could be halted, or even reversed, with an additional input of organic C into the soil at a minimum rate of 0.4 Mg ha^–1^ yr^–1^.

## Introduction

Cultivation of natural soils generally leads to a reduction of soil organic carbon (SOC) because cultivation enhances the rates of carbon (C) mineralization, reduces the amount of biomass C returned to the soil, and accelerates SOC erosion and leaching processes [1], [2]. SOC loss can be slowed, or even reversed, by optimizing agricultural management (e.g., stubble retention, fertilization and conservation tillage), thereby not only mitigating climate change but also improving soil fertility [2], [3].

Australian cropland has experienced tremendous loss of SOC since the European settlement began, due largely to the loss of above-ground biomass C after the conversion of native land for agriculture [4]. Recently, a national meta-analysis showed that SOC stores in the top meter of Australian agricultural soils have decreased by 40 to 60% compared with pre-clearing levels [5]. Based on a regional meta-analysis, Dalal and Chan [6] suggested that, in the Australian wheat belt, soil would sequester a large amount of atmospheric CO_2_ 20 years after the adoption of improved management techniques. However, cropland SOC dynamics depend on a balance between C production and decomposition and are regulated not only by management but also by highly variable climate and soil conditions [5], [7]. A regional survey, based on field experiments, reported large uncertainties in SOC estimates due to the spatiotemporal variability of the study sites. Accurately assessing regional SOC dynamics is difficult, particularly in Australian croplands, due to the lack of detailed climate, soil, and management-related information.

**Figure 1:**
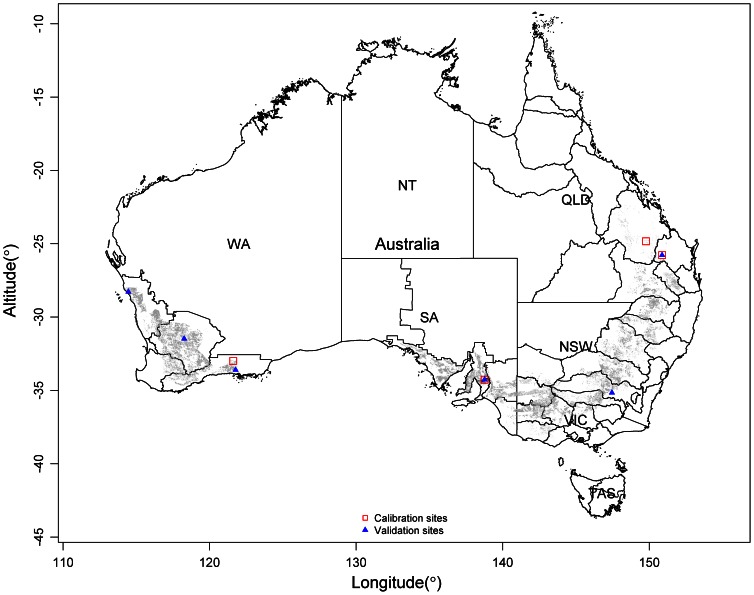
Spatial distribution of wheat-growing areas across the Australian wheat belt and locations of long-term experiments. Wheat is planted in the grey areas, red open squares show the sites for model calibration and blue solid triangles show the sites for model validation. NT, QLD, NSW, VIC, SA, WA and TAS refer to Northern Territory, Queensland, New South Wales, Victoria, Southern Australia, Western Australia and Tasmania, respectively.

Wheat-farming lands constitute approximately 40% of all Australian agricultural areas [8] and are distributed across several natural resource management (NRM) regions, primarily on the mainland, in a narrow crescent known as the wheat belt (Figure 1). Due to the adoption of modern cultivars and improved management practices, Australian wheat yields per unit area have increased significantly during the past several decades, especially from 1960 to 2010, during which time yields approximately doubled [9]. This increased wheat production has resulted in an enhanced amount of wheat residues [10]. Since the 1960s, improved management techniques (such as conservation tillage and stubble retention) have been widely adopted across many parts of the wheat belt to reduce the risk of soil and water erosion [11], [12], [13], [14]. Both the improvement of wheat production and the promotion of stubble retention are likely to cause an increase in SOC accumulation or a slow-down of SOC loss. Nevertheless, to the best of our knowledge, no comprehensive study has been conducted on large-scale cropland SOC change across the Australian wheat-growing areas.

It is widely recognized that a modeling approach has advantages when estimating spatiotemporal changes in SOC [1]. Dynamics of agricultural SOC are generally well captured by process-based models such as DNDC [15], RothC [16] and CENTURY [17], which have already been widely used to estimate changes in SOC at both national and continental scales [18], [19], [20]. More recently, Huang *et al.* [7] developed a biogeophysical model, Agro-C, which has been used to assess the national long-term agricultural SOC change in China [1]. The model consists of two sub-models: Crop-C for simulating crop net primary production, and Soil-C for computing SOC change. Previous validation studies indicate that the Soil-C sub-model properly simulates the observed changes in SOC in most cases across Chinese croplands [1], [7], [21], [22], [23].

Determining the regional cropland SOC dynamics would not only provide a better understanding of the factors and processes regulating C cycling and balance in the agro-ecosystems but would also provide insight into the effectiveness of methods for enhancing soil quality and plant production, as well as reducing greenhouse gas emissions. Our objective in this study is to quantitatively estimate regional-scale spatiotemporal changes in SOC across Australian wheat-growing areas between 1960 and 2010, so that policy-makers can make sensible, region-oriented decisions regarding SOC management.

## Materials and Methods

### 1.1 Model Calibration and Validation

#### 1.1.1 Model calibration

Agro-C is a biogeophysical model for simulating regional carbon budgets of agro-ecosystems on a large scale initially developed by Huang *et al.* [7]. The model consists of two submodels, Crop-C and Soil-C. Crop-C simulates processes involved with crop photosynthesis, autotrophic respiration, and net primary production (NPP). Soil-C simulates soil heterotrophic respiration via the decomposition of both input organic C (e.g., crop residues, roots and manure) and soil organic carbon. Changes in SOC are then determined by balancing the loss of soil carbon with the gain of input organic carbon. A detailed description of the Agro-C model was shown in Huang *et al.* [7]. The Soil-C submodel was recently modified by Yu *et al.* [1], who split the previous SOC pool into two sub-pools named light-C and heavy-C, with specific decomposition rates for each sub-pool. The light-C sub-pool is considered more biologically reactive, with turnover times ranging from a few months to a few years, while the heavy-C sub-pool is much more resistant to decomposition and can remain in the soil for decades or centuries [1]. Meanwhile, the C flow between different pools was also modified based on the following assumptions: (i) the decomposition of both the labile-C and the resistant-C converts a fraction of the C into the light-C pool, (ii) a fraction of the decomposed light-C is transferred into the heavy-C pool, and (iii) the decomposition of the heavy-C pool only produces CO_2_. Detailed structural modification of the Soil-C model was shown in Yu *et al.* [1].

In Soil-C, the parameters that need to be determined include the first-order reaction rates of light-C (*K_LC_*) and heavy-C (*K_HC_*), the fractions of the decomposed labile-C and resistant-C entering into the light-C pool (*F_LL_* and *F_RL_*, respectively), and the fraction of the decomposed light-C entering into the heavy-C pool (*F_LH_*). Other parameters, such as the first-order reaction rates of labile-C (*K_L_*) and resistant-C (*K_R_*), are taken from the original version of Soil-C [7]. The values of the five key parameters were initially set within a given range of intervals [1]. The final values of these parameters were determined by minimizing the mean deviation (see Eq. 5) between the simulated and observed SOC after running each combination of the five parameters on Soil-C using a parameter-space search method.

Measurements of SOC from long-term field experiments at four sites, including Brigalow, Tarlee, Warra and Salmon Gums (Figure 1 and Table S1), were used to calibrate Soil-C. The field experiment data used for this study were initially published by the Queensland Department of Primary Industry, South Australian Research and Development Institute, Queensland Department of Natural Resources and Mining, and CSIRO Land and Water. None of the field studies mentioned in this study involved endangered or protected species. In the four sites, annual mean temperature and precipitation ranged from 16.8 to 21.4°C and from 467 to 685 mm, respectively. At Brigalow, the original land use type was native forest; however, this site was cleared and converted to cropland in 1982. Following a 2-year fallow period, sorghum and wheat were planted in alternating years. Two cropping treatments, the continuous wheat system (CW) and the fallow-wheat system (FW), were adopted at the Tarlee site. The FW treatment was selected to for Soil-C submodel calibration, whereas the CW treatment was used for model validation. The site has been used for cereal production since 1935. In 1986, long-term field experiments were established covering various combinations of cropping systems and fertilization management. The wheat-lucerne (1 year, 2 years, respectively) rotation (WL, the first crop being wheat) was selected for model parameterization, and the lucerne-wheat (2 years, 1 year, respectively) rotation (LW, the first crop was lucerne) was selected for model validation. The Salmon Gums site, established in 1979, was parameterized for the Soil-C model using a continuous wheat treatment.

Table 1 shows the values of *K_LC_*, *K_HC_*, *F_LL_*, *F_RL_* and *F_LH_* in the calibrated Soil-C that resulted in a mean deviation of 0.002 MgC ha^−1^ between the simulated and the observed SOC at the four experimental sites (Figure S1). Generally, the above five parameters are within reasonable ranges. For example, the value of *K*_LC_ is smaller than the reaction rate of BIO (Microbial Biomass), and the value of *K*_HC_ is lower than the reaction rate of HUM (Humified Organic Matter) and higher than that of IOM (Inert Organic Matter) in the Roth-C model [16].

**Table 1:** Parameters in the original and modified Soil-C submodel.

Parameter	Original a	Modified a	Description
*K_LC_* (d^−1^)	2.5×10^−4^ (7.6)	2.5×10^−4^ (7.6)	First-order reaction rate of light-C pool
*K_HC_* (d^−1^)	1.8×10^−5^ (105.4)	2.6×10^−5^ (73)	First-order reaction rate of heavy-C pool
*F_LL_*	0.3	0.3	Fraction of decomposed labile-C to light-C
*F_RL_*	0.45	0.45	Fraction of decomposed resistant-C to light-C
*F_LH_*	0.45	0.3	Fraction of decomposed light-C to heavy-C

a Values in parentheses are the half-life residence time in years.

#### 1.1.2 Model validation

The calibrated Soil-C submodel was validated against independent datasets from 6 long-term experimental sites (Figure 1 and Table S1). These experimental data were derived from the National Carbon Accounting System Technical Report (*Integrated soils modeling for the National Carbon Accounting System No. 36*, available: http://www.pandora.nla.gov.au/tep/23322). At each site, crop biomass production was recorded at harvest every year, and SOC at a given profile depth was measured at both the start of the trials and after the harvest in some years. Table S1 shows the initial soil information at the start of each experiment.

For some experimental sites where SOC density was only reported in the top 10 cm (*SOC_0–10 cm_*) or 20 cm (*SOC_0–20 cm_*), the SOC density in the top 30 cm soil layer (*SOC_0–30 cm_*) was calculated using the SOC vertical distribution [24], [25], [26]:
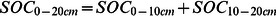
(1)

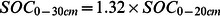
(2)

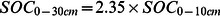
(3)where 1.32 and 2.35 are the conversion coefficients.

Following Yu *et al.* [1], three statistical criteria were used to evaluate the model performance: the root mean squared error (*RMSE*, Eq. 4) was calculated to measure the coincidence between the observed and the simulated SOC, the relative mean deviation (*RMD*, Eq. 5) was computed to evaluate the systematic bias of the model, and the model efficiency (*EF*, Eq. 6) was calculated to estimate model performance in relation to the observed mean. Linear regression analysis between simulated and observed SOC was also used to evaluate the model performance.



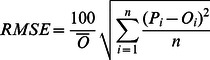
(4)

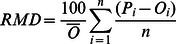
(5)

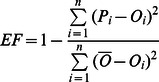
(6)where *P* and *O* represent the model estimates and the field measurements, respectively, 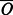 is the mean observed SOC, and *n* is the total number of observations.

### 1.2 Simulation of Changes in SOC

The SOC change was simulated using the Agro-C model because the soil moisture that affects SOC dynamics in the Soil-C submodel is computed via the Crop-C submodel in Agro-C [1], [7]. The changes in SOC were simulated with a daily step from 1960 to 2010. Up-scaling of the Agro-C model was accomplished by first computing the model inputs within each interested NRM region across the wheat-belt and then running the model in each of the NRM regions.

#### 1.2.1 Compilation of model inputs

In this study, the main input data used for the Agro-C model include climate, soil, farming management and crop yield (Table 2).

**Table 2:** Main input data for running the Agro-C model.

Category	Item (Unit)	Source (Reference)
Climate	Daily maximum and minimum temperature (°C), precipitation (mm), solarradiation (MJ m^−2^ d^−1^), wind speed (m s^−1^) and relative humidity (%)	SILO Patched Point Dataset (http://www.bom.gov.au/silo/)
Soil properties	Concentrations of organic carbon and total nitrogen (g kg^−1^), bulk density(g cm^−3^), sand and clay fraction (kg kg^−1^) and pH	Australian Soil Resource Information System (ASRIS) (http://www.asris.csiro.au/)
Farming managementand crop yield	Dates of planting, heading and harvest (dd/mm), timing (dd/mm) and rateof fertilizer N application (kg ha^−1^) and grain yield (kg ha^−1^)	Year Book of Australia (1960–2010, http://www.abs.gov.au/) Scott *et al*. [41]; Llewellyn and D’Emden [33]
Atmosphere	Atmospheric CO2 concentration	WMO World Data Centre for Greenhouse Gases (http://gaw.kishou.go.jp/wdcgg/)

Gridded daily climate data (i.e., maximum and minimum temperature, precipitation, relative humidity and radiation) from 1960 to 2010 with a spatial resolution of 0.05° × 0.05° were obtained from the Queensland Climate Change Centre of Excellence (http://ehp.qld.gov.au/index.html). These datasets are constructed by applying spatial interpolation algorithms to historical climate data from approximately 4,600 observations across Australia [27]. For each studied NRM region, the daily climate input data were calculated using the mean of the gridded climate data located within that specific NRM region.

Input soil properties include the concentrations of organic carbon and total nitrogen, bulk density, clay and sand fraction, and pH in the topsoil to 30 cm depth. These soil properties, with a 0.01° × 0.01° spatial resolution, were obtained from Australian Soil Resources Information System (ASRIS) [28]. These datasets represent some 164,030 soil profile measurements made since the 1960s across intensively used agricultural zones in Australia and are currently the best continental-scale soil profile data available to the public [29].

The spatial distribution of cropland was computed based on a national-scale land use map of Australia (Version 3 for 2005/2006), obtained from the Australian Bureau of Rural Sciences [30]. Due to a lack of yearly cropland data with sufficient spatial distribution from 1960 to 2010, we assumed that the area of cropland in the wheat-growing regions did not significantly change over this time period. The annual wheat yield at the NRM regional level from 1960 to 2010 was obtained from the Australian Bureau of Statistics (http://www.abs.gov.au/).

Carbon enters the soil through crop residue retention, root mass, and organic amendments (e.g., animal manure). Following Huang *et al*. [31], annual above-ground residue mass was calculated via the economic yield of the crop and the ratio of above-ground residue production to economic yield. The contribution from root mass was estimated as 40% of the crop residue mass, and all above-ground and root residues were assumed to have a C content of 45% [32]. The proportion of the above-ground residue that is retained in the soil across different regions was estimated based on the work of Llewellyn and D’Emden [33]. The rate of residue retention in different cropping Australian croplands over the study period has been uneven. In Queensland, the burning of crop residues has been almost abandoned, with approximately 95% of stubble being incorporated into soils or retained in the fields. In WA, approximately 17% of stubble has traditionally been burned. In NSW, Victoria and SA, however, burning was more common (24–35% of stubble burned), especially in NSW and Victoria. Due to a lack of yearly stubble retention rate data, we assume the stubble retention rate across different regions did not change significantly over time. Additionally, because the areas that have adopted animal manure application constitutes only 0.4% of the whole wheat belt [6] and there is no detailed information on the spatial distribution of these areas, we ignore the impacts of animal manure application on the regional cropland SOC change.

#### 1.2.2 Model initialization

In Agro-C, the half-life residence time for labile-C, resistant-C, light-C and heavy-C were 0.1 y, 2.3 y, 7.6 y, and 105.4 y [1], respectively. To obtain initial values for the different C pools and to initialize the Agro-C model, a ‘spin-up’ procedure was performed by running the model for one hundred years (i.e., 1900–2000). The initial fractions of light-C and heavy-C were assigned to be 0.25 and 0.75 [1], respectively. The input values for organic carbon were derived from annual crop yield. Climatic data from the same time period were used during the spin-up procedure.

#### 1.2.3 Uncertainty assessment

The two main factors that can influence SOC change are soil environment and carbon input [34], both of which can result in major uncertainties in the estimated SOC change.

For each initial soil-related model input (i.e., initial soil organic carbon and nitrogen concentrations, clay fraction and pH), the probability distribution function (PDF) for each NRM region was calculated based on the grid values within that region. We also used a Monte Carlo analysis to develop the PDF for carbon input within the ranges of mean±10% for each NRM region. The calibrated and validated Agro-C model was then run 300 times in each NRM region with the initial soil properties and carbon input randomly assigned from the PDFs of soil-related covariates and carbon input, respectively. Finally, the model output was analyzed to produce an empirical distribution of SOC changes on a regional level [1], [20]. A 95% confidence interval of SOC change was also estimated based on the 500 simulations in each NRM region. All the above analyses were conducted using the R version 2.15.1 software package [35].

## Results

### 2.1 Model Performance

**Figure 2:**
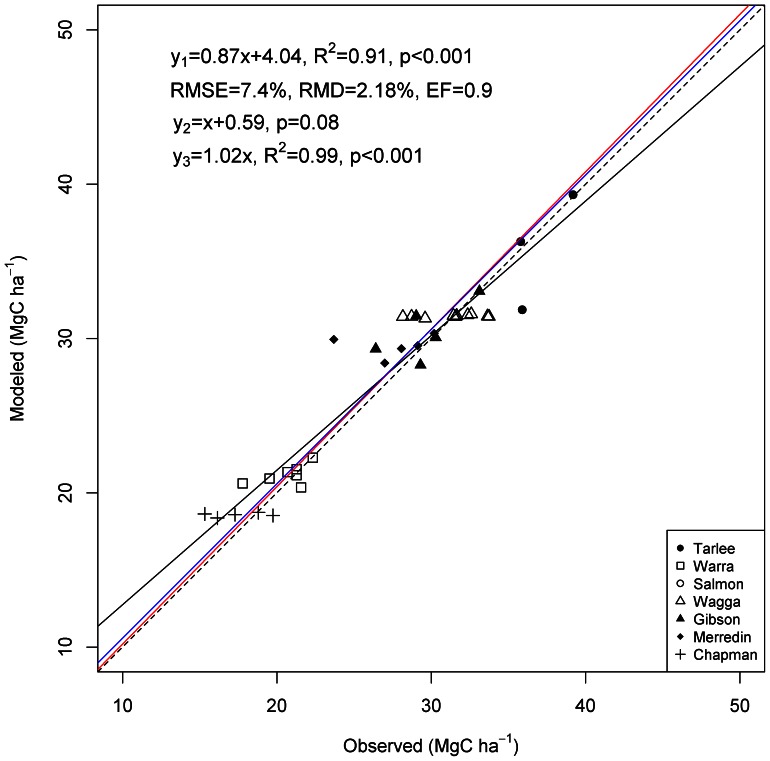
Modeled vs. observed SOC at the validation sites. Dashed line is 1∶1. Black solid line (y_1_) shows the linear regression analysis, blue solid line (y_2_) shows the statistical tests on the intercept, and red solid line (y_3_) shows the statistical tests on the slope.

The calibrated Agro-C model, in general, captured SOC changes better than the original version of Agro-C at all of the calibration sites except Tarlee (Figure S1). The simulated trends in SOC change in the top 30 cm of soil at different validation sites generally agreed well with the observed data during the selected study period (Figure 2 and Figure S1), except some exceptional variations (e.g., observed SOC in 1993 at Warra and in 1986 at Merredin). In general, Agro-C simulations could explain more than 90% of the variation in the SOC when data from all validation sites were pulled together (Figure 2), indicating an overall good performance of the calibrated Agro-C model. Statistical tests on both the slope and the intercept indicated that, in general, Agro-C modeled SOC agreed well with corresponding observations (Figure 2).

### 2.2 Estimated Changes in SOC from 1960 to 2010

**Figure 3:**
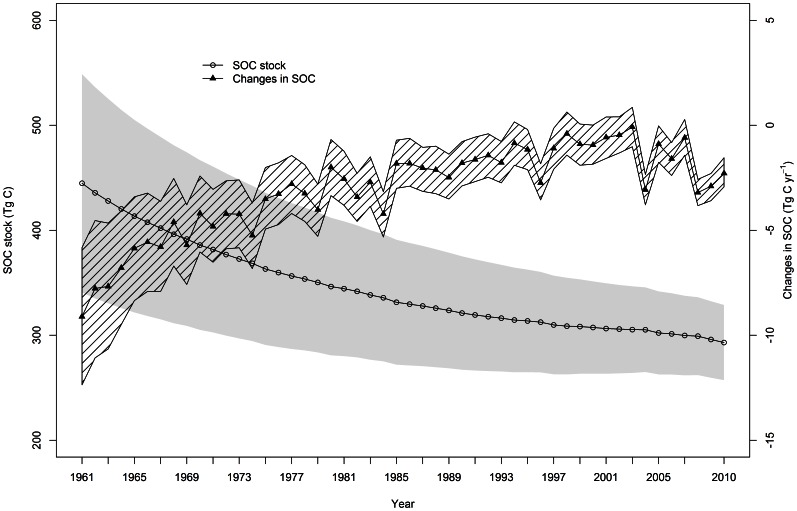
Simulated SOC levels and annual change in SOC of Australian wheat-growing land from 1960 to 2010. The open circles show the SOC levels and the solid triangles show the annual SOC change. The grey and shaded areas show the 95% confidence interval of SOC levels and annual SOC change, respectively.

Our Agro-C simulation results suggest that SOC in Australian wheat-growing areas has decreased over the last five decades (Figure 3). On average, the modeled SOC densities of the entire Australian wheat-growing areas in each decade were 27 (±22%, 95% confidence interval) Mg ha^−1^ during the 1960s, 24 (±17%) Mg ha^−1^ during the 1970s, 22 (±18%) Mg ha^−1^ during the 1980s, 21 (±14%) Mg ha^−1^ during the 1990s, and 20 (±15%) Mg ha^−1^ during the 2000s, respectively (Table 3). Although average SOC densities decreased over the study period, the rate of SOC loss is declining, with more SOC lost in the first 30 years than in the last 20 years (Table 3 and Figure 3).

**Table 3:** Estimated changes in SOC levels (0–30 cm) in different regions of Australian wheat-growing areas over the last 50 years.

						1960s						1970s				
Region	Area (Mha)		Initial SOC density(Mg C ha^−1^)			Average SOCdensity (Mg C ha^−1^)		Average C input (Mg C ha^−1^ yr^−1^)		Changes in SOC(Tg C)		Average SOC density(Mg C ha^−1^)		Average C input(Mg C ha^−1^ yr^−1^)		Changes in SOC(Tg C)
QLD	0.86		33 (22–43)			30 (21–39)		1.18		−4 (−6– −2)		26 (19–33)		1.13		−3 (−4– −1)
NSW	3.83		31 (21–42)			28 (19–38)		0.88		−19 (−28– −9)		25 (17–32)		0.93		−11 (−17– −6)
WA	5.28		29 (24–34)			26 (22–30)		0.86		−23 (−30– −17)		23 (20–26)		0.96		−13 (−17– −9)
SA	2.87		29 (23–34)			26 (21–31)		0.98		−11 (−14– −6)		24 (20–28)		1.01		−6 (−9– −4)
VIC	2.24		28 (24–33)			26 (22–30)		1.15		−7 (−9– −5)		24 (21–27)		1.31		−4 (−5– −2)
Wheat belt	15.08		30 (23–36)			27 (21–33)		0.95		−64 (−87– −39)		24 (19–28)		1.02		−37 (−52– −22)
**1980s**					**1990s**						**2000s**					**1960–2010**
**Average SOC density** **(Mg C ha^−1^)**		**Average C input (Mg C ha^−1^ yr^−1^)**		**Changes in SOC (Tg C)**	**Average SOC density (Mg C ha^−1^)**		**Average C input (Mg C ha^−1^ yr^−1^)**		**Changes in** **SOC (Tg C)**		**Average SOC** **density (Mg C ha^−1^)**		**Average C input (Mg C ha^−1^ yr^−1^)**		**Changes in** **SOC (Tg C)**	**Changes in SOC** **(Tg C)**
23 (17–29)		1.27		−2 (−3– −1)	21 (16–26)		1.14		−2 (−3– −1)		20 (15–23)		1.29		−1 (−2– −1)	−12 (−18– −6)
22 (16–28)		0.99		−7 (−12– −3)	21 (16–26)		1.31		−5 (−8– −1)		20 (16–24)		1.02		−6 (−9– −3)	−48 (−74– −22)
21 (18–24)		1.03		−10 (−13– −6)	20 (17–22)		1.48		−3 (−6–0)		19 (17–21)		1.40		−4 (−5– −2)	−53 (−71– −34)
22 (18–25)		1.10		−4 (−6– −3)	21 (18–24)		1.43		−2 (−3–0)		21 (18–23)		1.35		−3 (−4– −1)	−26 (−36– −14)
23 (21–25)		1.41		−2 (−4– −1)	22 (20–24)		1.40		−2 (−2– −1)		21 (20–23)		1.34		−2 (−3– −1)	−17 (−23– −10)
22 (18–26)		1.10		−25 (−38– −14)	21 (17–24)		1.40		−14(−22– −3)		20 (17–23)		1.28		−16 (−23– −8)	−156 (−222– −86)

Averaging for each decade, the rate of SOC loss was estimated to be approximately 6.4, 3.7, 2.5, 1.4 and 1.6 Tg yr^−1^ in the 1960s, 1970s, 1980s, 1990s and 2000s, respectively. Summing up the yearly changes in SOC over the period from 1960 to 2010, the loss of SOC in Australian wheat-growing areas was estimated to be 156 Tg C, with a range from 86 to 222 Tg C at the 95% confidence level (Figure 3). It is noteworthy that after the decline in the first few years, SOC seemed to reach a new, steady state in the mid-2000s, followed by a decrease in the following several years (Figure 3).

### 2.3 Spatial Characteristics of Estimated SOC Change

In general, changes in SOC density from 1960 to 2010 within different states are consistent with that of the entire Australian wheat-growing area. Nevertheless, there are differences in SOC density changes across different states. On average, compared with the initial status, SOC density during the 2000s decreased by 13 Mg ha^−1^ in Queensland, 11 Mg ha^−1^ in New South Wales, 10 Mg ha^−1^ in Western Australia, 8 Mg ha^−1^ in Southern Australia, and 7 Mg ha^−1^ in Victoria, respectively (Table 3).

**Figure 4:**
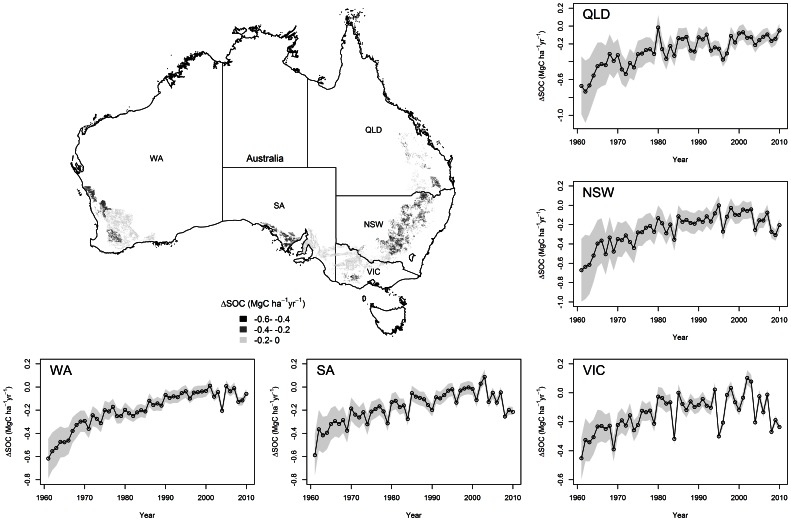
Estimated changes in SOC density (△SOC, Mg C ha^−1^ yr^−1^) in different regions of Australian wheat-growing areas. The shaded areas represent the lower and upper bounds of the estimates at 95% confidence level. QLD, NSW, VIC, SA and WA refer to Queensland, New South Wales, Victoria, Southern Australia and Western Australia, respectively.

Overall, SOC stocks in the topsoil to 30 cm depth were estimated to have decreased by 12 (±50%) Tg in Queensland, 48 (±54%) Tg in New South Wales, 53 (±34%) Tg in Western Australia, 26 (±38%) Tg in Southern Australia and 17 (±35%) Tg in Victoria (Figure 4 and Table 3).

## Discussion

**Figure 5:**
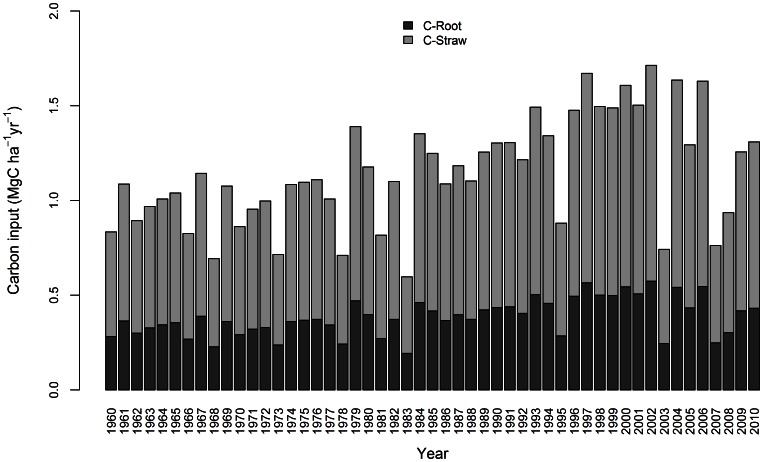
Average rates of carbon input from 1960 to 2010.

Our simulation results showed that although the Australian wheat-growing areas have in general experienced SOC loss from 1960 to 2010, the rate of SOC loss has declined over time (Figure 3 and Table3). Simulations indicated that approximately 80% of the loss of SOC across Australian wheat-growing areas occurred in the first 30 years, with a lower amount of SOC loss in the later 20 years. SOC loss appeared to reach a new steady-state during the first few years of the 2000s (Table 3 and Figure 3). The achievement of steady-state SOC level is attributed to two primary factors. First, enhanced crop production increased the amount of residue and root input into the soil (Figure 5), thus leading to a reduction in SOC loss over time. Second, following the initial rapid decline, SOC would reach a new steady-state after about 50 years of cultivation, therefore resulting in a slower rate of SOC loss during the later years [2], [36].

Model estimates suggested that SOC loss was slowed during the 1990s and 2000s, particularly between 1998 and 2003 when SOC loss almost halted (Figure 3). This cessation in SOC loss was likely due to relatively high C input rates, averaging 1.56 Mg C ha^−1^ yr^−1^, between 1997 and 2002 (Figure 5). However, SOC decreased significantly from 2003 to 2010 (Figure 3), when crop yields were low and C input averaged only 1.20 Mg C ha^−1^ y^−1^ (Figure 5). We suggest that the SOC loss across most of the Australian wheat-growing areas could be halted or even reversed with an additional input of organic carbon into the soil at a minimum rate of 0.4 Mg ha^–1^ yr^–1^, relative to the mean rate from 2003 to 2010.

Recently, using Agro-C, Yu et al. [1] modeled the SOC changes across Chinese agro-ecosystems during the last three decades and found that Chinese cropland SOC has generally increased. The differences in cropland SOC patterns in the two countries during the past several decades are mainly due to, first, the different initial SOC values. In generally, Chinese croplands have experienced a long history of intense cultivation, which led to a very low level of SOC [18], whereas the Australian soils have not been cultivated as intensively for as long. Second, the annual amount of organic C input in China [1] is generally higher than that in Australia, allowing for more carbon to be sequestrated in the soil. For example, SOC densities increased by 0.35 Mg C ha^−1^ yr^−1^ in south and central China but decreased by 0.18 Mg C ha^−1^ yr^−1^ in Victoria during the first 30 years of the simulations, even though their initial SOC densities were similar (approximately 28 Mg C ha^−1^ ). In contrast, the annual C input in Victoria was only 1.29 Mg ha^−1^ yr^−1^ (Table 3), approximately 38% of that in south and central China [1].

On average, the rate of SOC loss across Australian wheat-growing areas from 1960 to 2010 is 3.12 Tg y^−1^, approximately 4.8% of the total Australian fossil-fuel carbon emissions during the same period (http://cdiac.ornl.gov/trends/emis/aus.html). These results imply that although Australian agricultural soil acted as a net carbon source, its contribution to increasing atmospheric carbon dioxide is much smaller than that of fossil fuel production and consumption.

The net change in SOC is determined by the balance of C inputs by crop production and outputs by decomposition, both of which are regulated by both management and environmental factors. In this study, higher rates of SOC decreases were found in regions with relatively low annual precipitation and high annual temperature (climatic data not shown), such as Queensland, northern parts of New South Wales, and Western Australia (Figure 4 and Table 3). This can be attributed to the following factors: precipitation provides water to support crop growth and leads to more C input into soil through stubble retention, whereas lower temperatures can inhibit soil respiration, thereby reducing SOC decomposition rates [37]. Furthermore, historical land use also impacts long-term SOC change. For example, SOC declined more in Queensland, which has a relatively higher initial SOC level (Table 3) than other regions. This difference in initial conditions is due to the relatively short history of cultivation in Queensland compared to the other regions [38]. Consequently, a higher C input rate is needed in Queensland to balance SOC loss.

Another implication of our results is that during the last five decades, C input through retention of crop residues has failed to compensate for C output across Australian wheat-growing areas. Thus, soil has acted as a net C source and might therefore be contributing to global warming. For the purpose of accumulating SOC and reducing greenhouse gas emissions, future agricultural management methods need to be improved. For example, increasing rotation complexity (e.g., increasing crop diversity or introducing legumes into rotation) has been reported to significantly promote SOC accumulation when compared to single wheat farming systems [39]. Additionally, in many regions, nitrogen is a dominant limiting factor for crop growth [40]. Adopting nitrogen fertilization would inevitably enhance both crop production and SOC accumulation. Finally, addition of animal manure would not only supply nutrients for crop growth but would also directly add C into the soil [5].

## Supporting Information

Figure S1 **Comparison of simulated and observed SOC at different calibration sites.** Open circles show the observed values, dashed lines show the simulated values before model calibration, and solid lines show the simulated values after calibration.
(TIF)

Figure S2 **Comparison of simulated and observed SOC at different validation sites.** Open circles show the observed values and solid lines show the simulated values.
(TIF)

Table S1 **Locations and initial soil properties of the calibration and validation sites.**
(DOC)
