# Supplementary figures and images for: Modeling Soil Organic Carbon Change across Australian Wheat Growing Areas, 1960–2010
Source: PLoS One. 2013 May 16;8(5):e63324. doi: 10.1371/journal.pone.0063324 (PMC3656038; doi:10.1371/journal.pone.0063324)

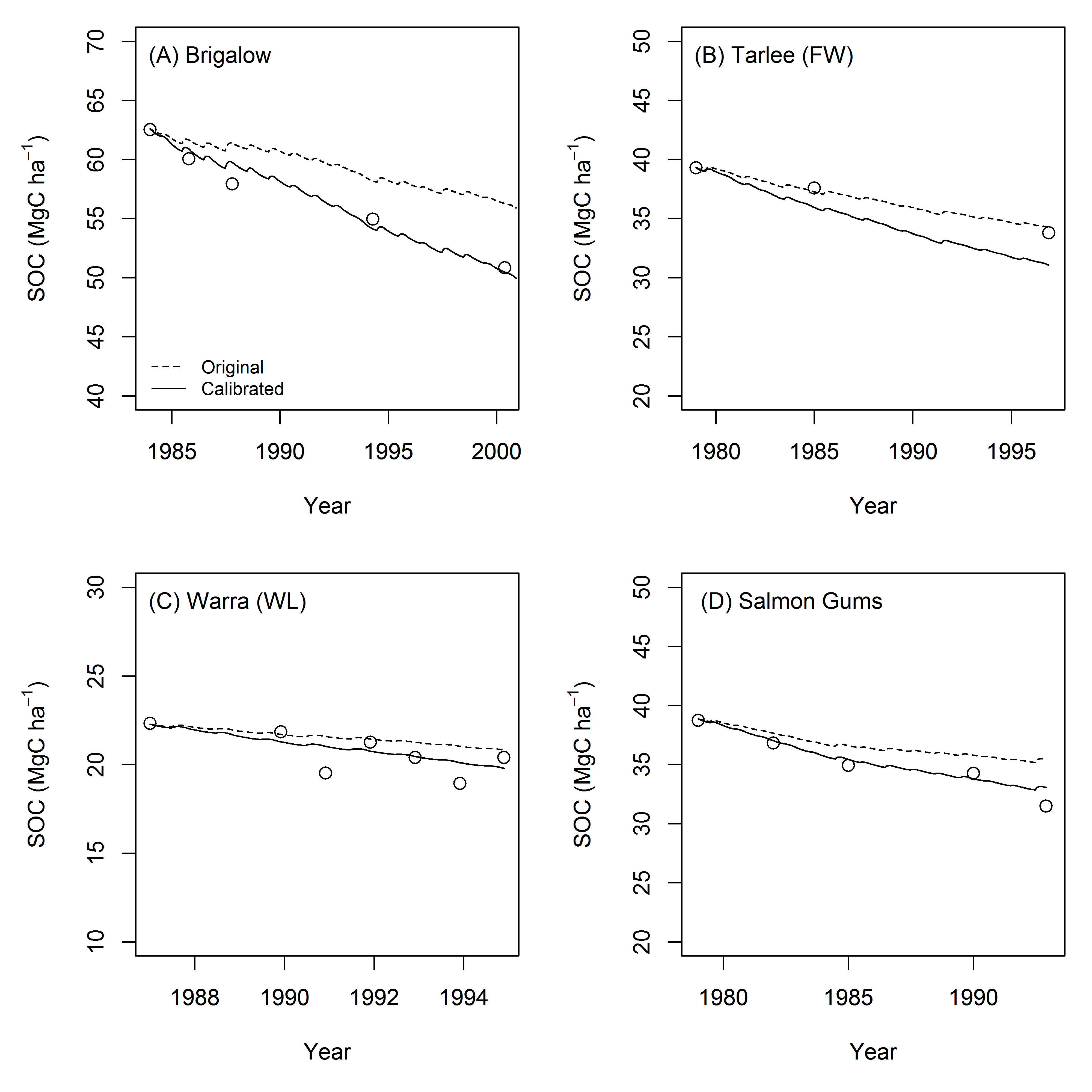

Supplement: Figure S1 — Comparison of simulated and observed SOC at different calibration sites. Open circles show the observed values, dashed lines show the simulated values before model calibration, and solid lines show the simulated values after calibration. (TIF) [file pone.0063324.s001.tif]

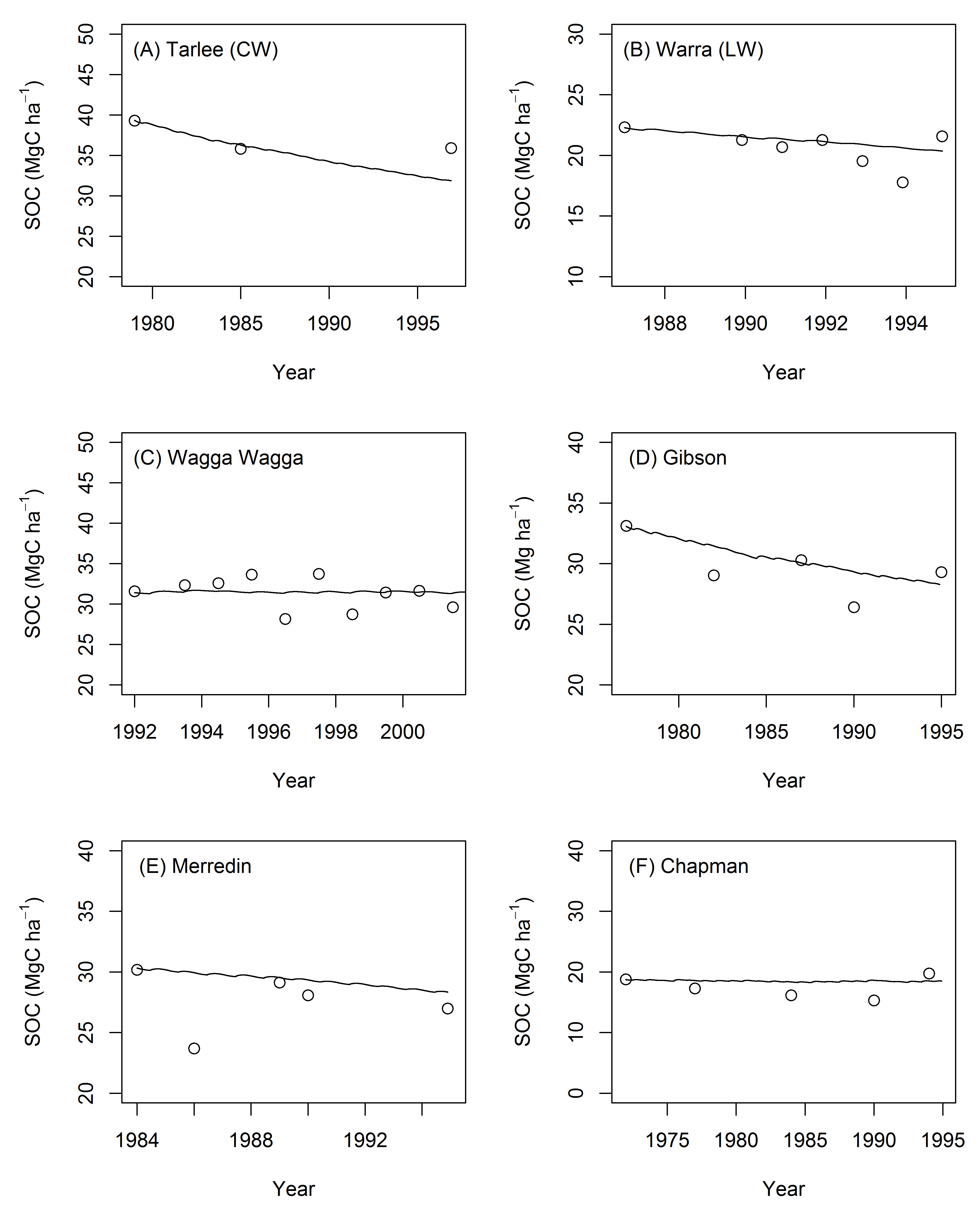

Supplement: Figure S2 — Comparison of simulated and observed SOC at different validation sites. Open circles show the observed values and solid lines show the simulated values. (TIF) [file pone.0063324.s002.tif]
