# Supplementary material for: Modeling Soil Organic Carbon Change across Australian Wheat Growing Areas, 1960–2010
Source: PLoS One. 2013 May 16;8(5):e63324. doi: 10.1371/journal.pone.0063324 (PMC3656038; doi:10.1371/journal.pone.0063324)
Supplement: Table S1 — Locations and initial soil properties of the calibration and validation sites. (DOC) [file pone.0063324.s003.doc]

Table S1. Locations and initial soil properties of the calibration and validation sites.

| Sites ^a^ | Latitude | Longitude | Altitude | Study period | Initial soil properties of top 20 cm profile | | | | | |
| --- | --- | --- | --- | --- | --- | --- | --- | --- | --- | --- |
|  |  |  |  |  | Sand (%) | Clay (%) | Bulk density (g cm^-3^) | pH | SOC (%) | Total N (%) |
| Brigalow | -24.83 | 149.78 | 177 | 1984-2000 | 45 | 38 | 1.33 | 6.9 | 1.81 | 0.14 |
| Tarlee | -34.28 | 138.77 | 200 | 1979-1996 | 60 | 22 | 1.2 | 6.9 | 1.26 | 0.10 |
| Warra | -26.78 | 150.88 | 320 | 1987-1994 | 27 | 56 | 1.26 | 8.7 | 0.68 | 0.06 |
| Salmon Gums | -32.99 | 121.62 | 259 | 1979-1992 | 36 | 28 | 1.3 | 8.6 | 1.15 | 0.10 |
| Wagga Wagga | -35.16 | 147.46 | 184 | 1992-2002 | 56 | 29 | 1.51 | 5.9 | 0.8 | 0.08 |
| Chapman | -28.28 | 114.46 | 123 | 1972-1994 | 45 | 10 | 1.47 | 6.5 | 0.49 | 0.04 |
| Gibson | -33.61 | 121.78 | 151 | 1977-1994 | 55 | 3 | 1.59 | 6 | 0.80 | 0.07 |
| Merredin | -31.48 | 118.27 | 317 | 1984-1994 | 57 | 20 | 1.62 | 5.7 | 0.72 | 0.05 |

^a^ Data of FW treatment at Tarlee and WL treatment at Warra, and data of Brigalow and Salmon Gums were used to calibrate the model, while data of CW treatment at Tarlee and LW treatment at Warra, and data of Wagga Wagga, Chapman, Gibson and Merredin were used to validate the model.
